# New Smart Sensor for Voltage Unbalance Measurements in Electrical Power Systems

**DOI:** 10.3390/s22218236

**Published:** 2022-10-27

**Authors:** Santiago Bogarra, Jaime Saura, Alejandro Rolán

**Affiliations:** 1 Department of Electrical Engineering, Technical University of Catalonia, 08222 Terrassa, Spain; 2 Department of Automatic Control, Technical University of Catalonia, 08019 Terrassa, Spain

**Keywords:** smart sensor, voltage sensor, voltage unbalance, voltage measurement, power quality

## Abstract

This paper deals with voltage unbalances and how they can be quantified according to the standards. Firstly, a comparison between the different unbalance voltage factors is conducted in order to remark on their divergences. Secondly, according to the standard that better represents the phenomenon, i.e., EN 50160, a new methodology is proposed to quantify the voltage unbalance factor (VUF). In order to do so, it is recommended to measure the voltage unbalance in three-phase installations by means of a new smart sensor based on a single voltage sensor, which measures the direct-current (DC) voltage at the output of a three-phase diode bridge rectifier, while current methods make use of three voltage sensors (which can measure either phase-to-neutral voltages or phase-to-phase voltages). Furthermore, both simulation and experimental results have been carried out to validate the proposed methodology. Finally, a new voltage unbalance factor (and the corresponding methodology to obtain it from the measured DC voltage) is proposed.

## 1. Introduction

Electrical and electronic equipment can work correctly if the supply voltage is within a specific interval around the nominal value [1]. In three-phase power systems, the ideal supply voltages are sinusoidal and balanced, i.e., the three phases have the same root mean square (RMS) values, and their phase difference is 2π/3 rad. However, in a real scenario, the power system voltages are usually unbalanced, especially in the low voltage distribution network, where the unequal distribution of single-phase loads can cause such unbalance [2]. Nevertheless, other causes exist, such as the asymmetry of the impedances of the transformer windings or blown fuses in capacitor banks. Let us consider the example of the voltage unbalance rate in power systems: in the USA, 66% of electric distribution systems have less than 1% voltage unbalance, 98% less than 3%, and 2% more than 3% [3].

According to the IEC 60038 standard [4], two different voltages in electrical networks and installations are distinguished. The first is the supply voltage between phases, or between a phase and neutral at the point of common coupling (PCC). The second is the service voltage between phases, or between phase and neutral at the terminal of the electrical device. The EN 50160 standard analyses the requirements for the supply voltage in distribution systems [1]. This standard defines voltage unbalance as a condition in which the RMS fundamental values of the phase-to-phase voltages, or the phase angles between consecutive phases of a three-phase system, are unequal. The same definition can be found in the IEC 61000-4-27 standard [5]. However, it should be noted that IEC 61000-4-27 refers to the service voltage, while EN 50160 considers the supply voltage.

One electrical power quality phenomenon that occurs most frequently in electrical installations is voltage unbalance. Even a small voltage unbalance at the transformer level can significantly disturb the current waveform on the connected loads, so it is necessary to quantify the voltage unbalance using unbalance factors [1,6,7,8]. Under normal operating conditions, excluding the periods with interruptions, the voltage variations should not exceed ±10% of the rated voltage, and during each one-week period, 95% of the 10-min averaged RMS values of the fundamental negative phase-sequence component of the supply voltage should be between 0% and 2% of the fundamental positive phase-sequence component [1]. Other factors, such as the ones defined by IEC 61000-4-30 [6] or by NEMA-G1 [7], avoid calculating the symmetrical components of the three-phase voltages. Some works in the literature propose a formula to calculate the voltage unbalance factor by using the phase-to-phase voltages directly [9,10,11]. A comparison between the different definitions of voltage unbalance is developed in [12].

Regarding induction motors, the negative sequence voltage unbalance factor indicates the level of voltage that is trying to run the motor in the opposite direction to that established by the positive sequence voltage. Unbalanced voltages can affect the motor behavior with unbalanced currents, which can cause an increase in the windings temperature (thus increasing copper losses), torque pulsations and vibrations, and a decrease in torque and power delivered, efficiency reduction and a drop in operational life [13,14,15,16]. Some studies in the literature show that the unbalance factor is not enough to predict the effects of voltage unbalance in motors because different types of unbalance have the same factors [17,18]. Therefore, additional information is required, such as the angle of the unbalance factor, thus defining the complex voltage unbalance factor [18,19,20], or the sequence voltages [17,21]; a new definition of voltage unbalance using supply phase shift is presented in [22].

Furthermore, it should be noted that voltage unbalance affects not only motors, but also power converters and drives, as shown in [23,24]. Therefore, it is essential to identify voltage unbalance problems in order to undertake corrective measures, for both the utility and the consumer. In addition, it is necessary to consider that voltage unbalance factors are influenced by the method that is used for calculating the RMS voltages, sampling windows size of the voltage waveform, and the sliding window method. It should be noted that the aforementioned definitions to quantify voltage unbalance [1,5,6,7,10,17,18,19,22] have limitations, so other alternative methodologies need to be defined to quantify voltage unbalance more appropriately [25]. Furthermore, although the unbalance factor can be obtained from the information available in smart meters [26,27], the development of new smart sensors is crucial to calculate the grid unbalance with greater accuracy. This paper tries to shed some light on this issue by means of:

1. Proposing a new methodology to quantify the voltage unbalance based on measuring the DC voltage at the output of three-phase diode bridge rectifiers, instead of the three alternating current (AC) voltages (phase-to-neutral or phase-to-phase voltages), as currently used in the literature.
2. Proposing a new definition of voltage unbalance factor based on time, considering the measured DC voltage, unlike existing methods in the literature, which propose the phase-to-neutral or phase-to-phase voltage measurements.

This paper is structured as follows: Section 2 exposes and discusses the definitions of voltage unbalance factors according to the standards. Section 3 proposes a new methodology to obtain the voltage unbalance factor, by means of the DC voltage measurement. Section 4 validates the proposed methodology with both simulation and experimental results. Section 5 proposes a new voltage unbalance factor. Finally, the conclusions of this work are drawn in Section 6.

## 2. Definitions of Voltage Unbalance Factors

The definitions of voltage unbalance factors according to the standards are described in this section. One or other definitions can be chosen depending on the available measurements, i.e., phase-to-neutral voltages or phase-to-phase voltages.

### 2.1. Ratio between the Negative-Sequence Component and the Positive-Sequence Component

The voltage unbalance factor (VUF) quantifies voltage unbalance in three-phase systems, which makes use of the symmetrical components of a three-phase unbalanced system. According to EN 50160 [1], IEEE Std. 1159 [2] and IEC 61000 [5,6], the VUF is defined by means of the following equation:

(1) $$\mathrm{VUF}=\frac{U_{2}}{U_{1}}\cdot 100,$$

where *U*_2_ is the modulus (RMS value) of the negative-sequence voltage phasor and *U*_1_ is the modulus (RMS value) of the positive-sequence voltage phasor. The complex sequence components can be calculated using the phase voltages by means of the *Fortescue* transformation [28], as follows:

(2) $${\bar{U}}_{1}=\frac{1}{3}\cdot \left({\bar{V}}_{a}+a\cdot {\bar{V}}_{b}+a^{2}\cdot {\bar{V}}_{c}\right);\;{\bar{U}}_{2}=\frac{1}{3}\cdot \left({\bar{V}}_{a}+a^{2}\cdot {\bar{V}}_{b}+a\cdot {\bar{V}}_{c}\right),$$

where ${\bar{V}}_{a},{\bar{V}}_{b},{\bar{V}}_{c}$ are the phase voltage phasors and $a=e^{j\frac{2\pi }{3}}$. Note that the *Fortescue* transformation makes it possible to convert a three-phase unbalanced system into the following three-phase balanced systems: positive sequence (with the same phase sequence as the initial unbalanced system); negative sequence (with the opposite phase sequence as the initial unbalanced system); and zero sequence (with all voltages in phase). Note also that there is no zero sequence for isolated wye or delta connection.

The CIGRE method [9], which is included in IEEE Std. 1159 [2], obtains the voltage unbalance factor without the need to calculate the voltage sequence components; it uses only the phase-to-phase voltage measurements:

(3) $${\mathrm{VUF}}^{\prime }=\sqrt{\frac{1-\sqrt{3-6\beta }}{1+\sqrt{3-6\beta }}}\cdot 100,$$

with *β* being a coefficient that depends on the phase-to-phase voltages, as follows:

(4) $$\beta =\frac{V_{\mathrm{ab}}{}^{4}+V_{\mathrm{bc}}{}^{4}+V_{\mathrm{ca}}{}^{4}}{{\left(V_{\mathrm{ab}}{}^{2}+V_{\mathrm{bc}}{}^{2}+V_{\mathrm{ca}}{}^{2}\right)}^{2}}\;,$$

where *V*_ab_, *V*_bc_, *V*_ca_ are the moduli (RMS values) of the phase-to-phase voltage phasors

### 2.2. Ratio between the Maximum Phase-to-Phase Voltage Deviation from the Average Phase-to-Phase Voltage and the Average Voltage

NEMA MG1 [7] defines the line voltage unbalance ratio (LVUR) as the ratio between the maximum phase-to-phase voltage deviation from the average phase-to-phase voltage and the average voltage, by means of the following equation:

(5) $$\mathrm{LVUR}=\frac{\max\left\{\left|V_{\mathrm{ab}}-V_{\mathrm{line}\_\mathrm{avg}}\right|,\left|V_{\mathrm{bc}}-V_{\mathrm{line}\_\mathrm{avg}}\right|,\left|V_{\mathrm{ca}}-V_{\mathrm{line}\_\mathrm{avg}}\right|\right\}}{V_{\mathrm{line}\_\mathrm{avg}}}\cdot 100,$$

with *V*_line_avg_ being the average value of the phase-to-phase voltages, as follows:

(6) $$V_{\mathrm{line}\_\mathrm{avg}}=\frac{V_{\mathrm{ab}}+V_{\mathrm{bc}}+V_{\mathrm{ca}}}{3},$$

where *V*_ab_, *V*_bc_, *V*_ca_ are the moduli (RMS values) of the phase-to-phase (line) voltage phasors.

### 2.3. Ratio between the Maximum Phase Voltage Deviation from the Average Phase Voltage and the Average Voltage

IEEE Std. 141 [29] defines the phase voltage unbalance ratio (PVUR) as the ratio between the maximum phase voltage deviation from the average phase voltage and the average voltage, by means of the following equation:

(7) $$\mathrm{PVUR}=\frac{\max\left\{\left|V_{a}-V_{\mathrm{phase}\_\mathrm{avg}}\right|,\left|V_{b}-V_{\mathrm{phase}\_\mathrm{avg}}\right|,\left|V_{c}-V_{\mathrm{phase}\_\mathrm{avg}}\right|\right\}}{V_{\mathrm{phase}\_\mathrm{avg}}}\cdot 100,$$

with *V*_phase_avg_ being the average value of the phase-to-neutral voltages, as follows:

(8) $$V_{\mathrm{phase}\_\mathrm{avg}}=\frac{V_{a}+V_{b}+V_{c}}{3},$$

where *V*_a_, *V*_b_, *V*_c_ are the moduli (RMS values) of the phase voltage phasors.

### 2.4. Ratio between the Maximum Phase Voltage Deviation from the Minimum Phase Voltage and the Average Voltage

IEEE Std. 936 [30] defines the phase voltage unbalance ratio as the ratio between the maximum phase voltage deviation from the minimum phase voltage and the average voltage, by means of the following equation:

(9) $${\mathrm{PVUR}}^{\prime }=\frac{\max\left\{V_{a},V_{b},V_{c}\right\}-\min\left\{V_{a},V_{b},V_{c}\right\}}{V_{\mathrm{phase}\_\mathrm{avg}}}\cdot 100,$$

where *V*_a_, *V*_b_, *V*_c_ are the moduli (RMS values) of the phase voltage phasors and *V*_phase_avg_ is calculated by Equation (8). Note that this factor is named PVUR’ in this paper in order to differentiate it from the PVUR obtained by means of Equation (7).

### 2.5. Discussion about the Definitons of Voltage Unbalance

Table 1 shows the quantification of different types of voltage unbalance by means of the symmetrical components, i.e., according to the definition given in the EN 50160 standard [1], which defines the voltage unbalance factor (VUF) [see Equation (1)]. The phasor diagram for each unbalance voltage scenario is given in the first column. The phasor expressions related to each phasor diagram of the first column are shown in the second column. In the third and fourth columns, the positive-sequence and negative-sequence voltage phasors, respectively, are given for each unbalance voltage scenario by means of applying Equation (2) to the phasor expressions of the second column. Finally, the VUF is calculated for each unbalance voltage scenario, by applying Equation (1) in per unit to the positive- and negative-sequence voltage phasors of the previous two columns. It should be noted that the parameter *h* represents the remaining voltage with respect to rated voltage (i.e., 1 = no voltage drop and 0 = 100% voltage drop).

It should also be noted that one of the drawbacks of the VUF is that under different voltage unbalance scenarios, the VUF is the same (see the 3rd and the 4th cases and last 3 cases in Table 1, though their phasor diagrams are different). This drawback is overcome in this paper by proposing a new definition of voltage unbalance (see Section 4).

Table 2 shows the quantification of different types of voltage unbalance by means of phase-to-phase voltages (i.e., according to NEMA MG1 [7]) and phase-to-neutral voltages (i.e., according to IEEE Std. 141 [29] and IEEE Std. 936 [30]), respectively. The expressions shown in this table have been obtained by applying Equation (5) in per unit for LVUR (second column), Equation (7) in per unit for PVUR (third column) and Equation (9) for PVUR’ (last column) to the voltage phasor expressions shown in the second column of Table 1. It should be noted that the parameter *h* represents the remaining voltage with respect to rated voltage (i.e., 1 = no voltage drop and 0 = 100% voltage drop). It is important to note that under certain scenarios, the quantification of unbalance voltage ratios exhibit long expressions (such as in the last two rows of Table 2), while under other scenarios, these expressions are shorter (e.g., PVUR and PVUR’ in the second row and in the third row of Table 2).

## 3. Proposal of a New Methodology to Measure the Voltage Unbalance Factor (VUF) According to EN 50160

Measuring instruments often use the ratio between the maximum phase-to-phase voltage deviation from the average phase-to-phase voltage and the average voltage in order to determine voltage unbalance. However, the ratio between the negative-sequence component and the positive-sequence component of the measured voltages, i.e., the VUF (according to EN 50160 [1], as shown in Equation (1)) is preferred among all the definitions of voltage unbalance because it directly represents the phenomenon.

In order to quantify the VUF in three-phase installations, the current methods in the technical literature require three sensors for measuring either phase-to-neutral voltages or phase-to-phase voltages, as explained in Section 2. This section presents a new methodology to quantify the VUF by means of a new smart sensor, which only measures the DC voltage.

**Figure 1:**
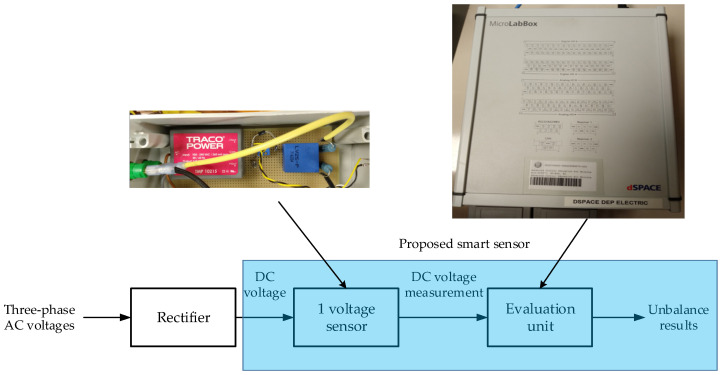
Block diagram with the proposed smart sensor to quantify the VUF.

Figure 1 shows the block diagram that compares the proposed smart sensor with the existing three-phase sensors. It is observed that the proposed smart sensor consists of the following two units: a voltage sensor (which measures the DC voltage in a rectifier) and an evaluation unit (which is able to compute the measured DC voltage in order to quantify the voltage unbalance). Note that the same idea is carried out in existing voltage sensors, but they need to measure three-phase voltages, while the proposed smart sensor measures just one voltage (DC voltage in the rectifier).

**Figure 2:**
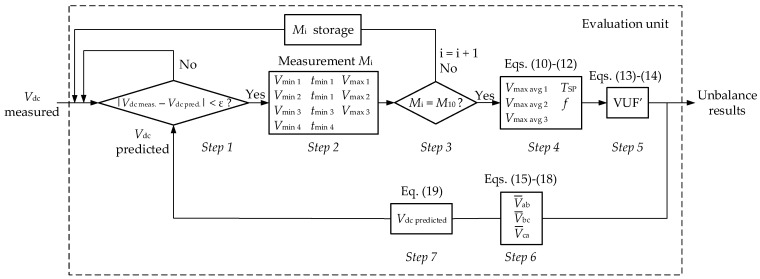
Flowchart with the proposed methodology to calculate the VUF (note that calculations are made within the evaluation unit of the proposed smart sensor, as shown in Figure 1).

Note that the evaluation unit shown in Figure 1 is responsible for calculating the VUF. The proposed methodology to be followed by the evaluation unit in order to obtain the VUF in three-phase installations is depicted in Figure 2. This methodology could be adapted in future works in order to obtain the unbalanced factors based on phase voltage measurements according to the standards discussed in Section 2.

**Figure 3:**
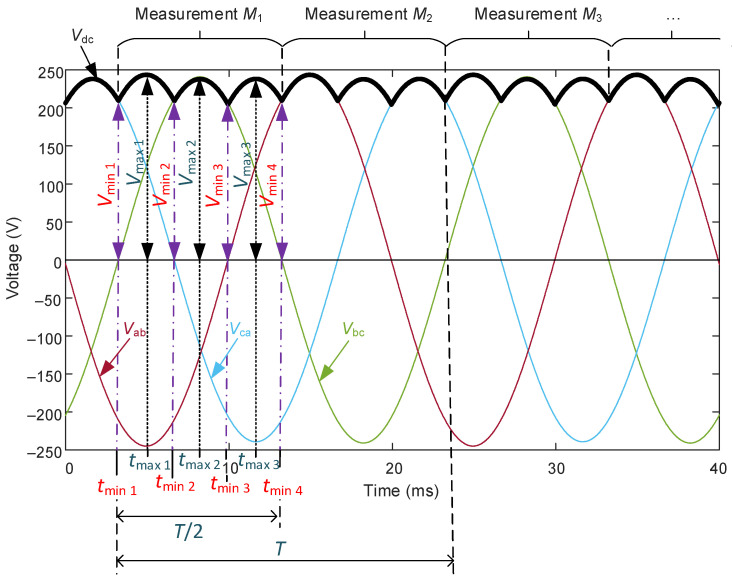
Voltage profile (phase-to-phase voltages and DC voltage) in a three-phase diode bridge rectifier with the following unbalanced voltage supply: phase-a voltage drop of 5% with respect to its rated value (100 V, 50 Hz). Determination of its first 3 consecutive maximum values and its first 4 consecutive maximum values, with their corresponding time values.

The methodology depicted in the flowchart of Figure 2 is detailed in the following seven steps:

1. To compare the measured DC voltage with the predicted DC voltage (the latter is obtained in Equation (19), step 7). If the difference between these values is lower than a predefined error ε, (in this application, an error ε = 5% has been considered), the measured value is valid and the method continues to step 2; otherwise, the measured value is not valid (e.g., due to a malfunction in the sensor, etc.) and a new comparison is made between the next DC measured value and the predicted DC value.
2. To compute 3 consecutive maximum values and 4 consecutive maximum values in the DC voltage measurement, with their corresponding time values. Figure 3 depicts this idea by showing the voltage profile that corresponds to the DC side of the rectifier when its AC side is fed with a one-phase voltage drop of 5% (in phase-a voltage) with respect to its rated value (100 V, 50 Hz). Note that the time lapse from the first minimum value to the fourth minimum value corresponds to a semi-period of the AC voltages (i.e., *t*_min 4_ − *t*_min 1_ = *T*/2). In Figure 3, the semi-period corresponds to 10 ms, because the rated frequency is 50 Hz.
3. To repeat 10 times over time the measurement explained in step 2 (so *M*_1_, *M*_2_, … *M*_10_ measurements are obtained). Table 3 shows the 10 measurements that correspond to the DC voltage profile depicted in Figure 3. It should be noted that every time that a measurement *M*_i_ is made, it is stored in the memory of the smart sensor, and every 5 cycles this table is fully updated with 10 new measurements.
4. To determine the average maximum values of the DC voltage (*V*_max 1_, *V*_max 2_, *V*_max 3_) as:

(10) $$V_{\max\mathrm{avg}\;i}=\left(\overset{M_{10}}{\underset{M_{1}}{\sum }}V_{\max\;i}\left(M_{j}\right)\right)/10,\;i=1,\;2,\;3;\;j=1,\;2,\ldots ,\;10.$$

In this step the semi-period value (*T*_SP_) is also obtained. Note that it corresponds to the difference between *t*_min 4_ and *t*_min 1_ for each measurement (as explained step 2 and shown in Figure 3). Since there are 10 measurements (see Table 3), then the semi-period *T*_SP_ is obtained as the average value of all semi-periods calculated for each measurement:

(11) $$T_{\mathrm{SP}}=\left(\overset{M10}{\underset{M1}{\sum }}\left(t_{\min4}\left(M_{i}\right)-t_{\min1}\left(M_{i}\right)\right)\right)/10,\;i=1,\;2,\ldots ,\;10.$$

The last calculation in this step is the grid frequency. Note that frequency is the inverse of the period (*f* = 1/*T*). Given that the semi-period value is known (it is obtained by Equation (11) and it corresponds to *T*_SP_ = *T*/2, then the grid frequency can be obtained as:

(12) $$f=\frac{2}{T_{\mathrm{SP}}}.$$

5. To obtain the VUF by means of Equation (3), though *β* is not obtained from the phase-to-phase voltages (as shown in Equation (4)), but from the maximum values obtained in step 4, according to Equation (10), so:

(13) $${VUF}^{\prime }=\sqrt{\frac{1-\sqrt{3-6\beta }}{1+\sqrt{3-6\beta }}}\cdot 100,$$

with:

(14) $$\beta =\frac{{\left(V_{\max\mathrm{avg}\;1}\right)}^{4}+{\left(V_{\max\mathrm{avg}\;2}\right)}^{4}+{\left(V_{\max\mathrm{avg}\;3}\right)}^{4}}{{\left[{\left(V_{\max\mathrm{avg}\;1}\right)}^{2}+{\left(V_{\max\mathrm{avg}\;2}\right)}^{2}+{\left(V_{\max\mathrm{avg}\;3}\right)}^{2}\right]}^{2}}$$

**Figure 4:**
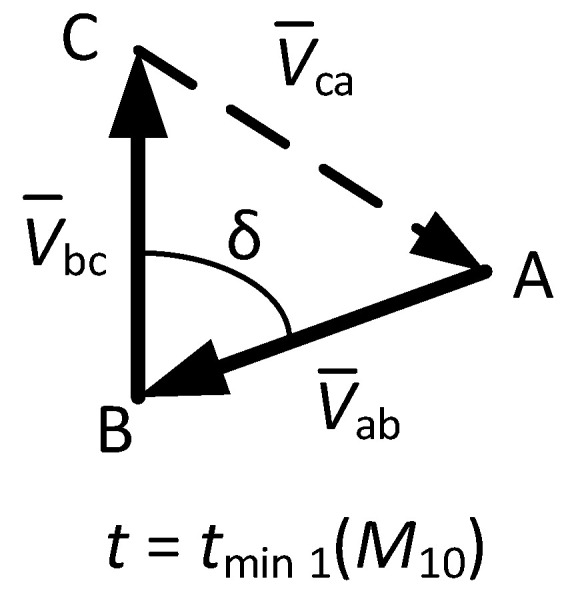
Phasor diagram of the reconstructed phase-to-phase voltages corresponding to the DC voltage profile shown in Figure 3, drawn at *t* = *t*_min 1_ (in measurement *M*_10_).

6. To reconstruct the phase-to-phase voltage phasors in *t*_min 1_ (from the last measurement, *M*_10_). Figure 4 shows the phasor diagram of phase-to-phase voltages, i.e., ${\bar{V}}_{\mathrm{ab}},{\bar{V}}_{\mathrm{bc}},\;{\bar{V}}_{\mathrm{ca}}$ at *t*_min 1_ (*M*_10_). It should be pointed out from this figure that the phasor ${\bar{V}}_{\mathrm{bc}}$ has an angle of π/2 rad, which matches with Figure 3, where the time evolution of the phase-to-phase bc voltage (cosine function) has an angle of π/2 rad at *t* = *t*_min 1_.

Then, ${\bar{V}}_{\mathrm{bc}}$ phasor has an angle of π/2 rad at *t*_min 1_ (*M*_10_) and its modulus corresponds to *V*_max avg 2_ (see Figure 3), so it is possible to write:

(15) $${\bar{V}}_{\mathrm{bc}}=V_{\max\mathrm{avg}\;2}\cdot e^{j\left(\frac{\pi }{2}\right)\;}.$$

Next, ${\bar{V}}_{\mathrm{ab}}$ phasor can be obtained by considering that at *t*_min 1_ (*M*_10_) its modulus corresponds to *V*_max avg 3_ and its angle equals –π/2—δ (see Figure 3 and Figure 4), so:

(16) $${\bar{V}}_{\mathrm{ab}}=V_{\max\mathrm{avg}\;3}\cdot e^{j\left(-\frac{\pi }{2}-\;\delta \right)\;}.$$

Note that the value of δ can be obtained by applying the law of cosines to the triangle made by the phase-to-phase voltage phasors shown in Figure 4, so:

(17) $$\delta =\mathrm{acos}\left(\frac{{\left(V_{\max\mathrm{avg}\;3}\right)}^{2}+{\left(V_{\max\mathrm{avg}\;2}\right)}^{2}-{\left(V_{\max\mathrm{avg}\;1}\right)}^{2}}{2\;V_{\max\mathrm{avg}\;2}\;V_{\max\mathrm{avg}\;3}}\right).$$

Finally, given that the sum of the phase-to-phase voltage phasors shown in Figure 4 is zero, it is possible to obtain the phasor ${\bar{V}}_{\mathrm{ca}}$ as:

(18) $${\bar{V}}_{\mathrm{ca}}=-{\bar{V}}_{\mathrm{bc}}-{\bar{V}}_{\mathrm{ab}}.$$

7. To obtain the predicted DC voltage by means of the *shadow-projection* method, which is defined in [31,32]. This method consists of obtaining the DC voltage in a three-phase diode bridge rectifier by means of the phase-to-phase voltages, according to the following formula:

(19) Vdc predicted(t′)=|Re{V¯ab·ej(2πft′)}|+|Re{V¯bc·ej(2πft′)}|+|Re{V¯ca·ej(2πft′)}|2,

where *f* is the grid frequency, which is obtained by means of Equation (12), and *t*′ is the current time instant, where the prediction is made, subtracting *t*_min 1_ in measurement *M*_10_:

(20) $$t^{\prime }=t_{\mathrm{current}}-t_{\min1}\left(M_{10}\right).$$

Finally, the methodology returns to step 1, in order to compare the predicted value of the DC voltage (Equation (19)) with the measured value by the sensor.

It is worth mentioning that the proposed methodology should be extended to the time interval proposed by current standards in order to quantify the voltage unbalance by the measuring devices, i.e., a minimum evaluation period of 1 week in time intervals of 10 min and/or 2 h, according to IEC 61000-4-30 [6].

## 4. Methodology Validation

The proposed methodology has been validated by both simulation and experimental tests in a laboratory setup. The authors consider that before being applied in industrial facilities, the proposed methodology should be validated in those facilities.

### 4.1. Simulation Results

**Figure 5:**
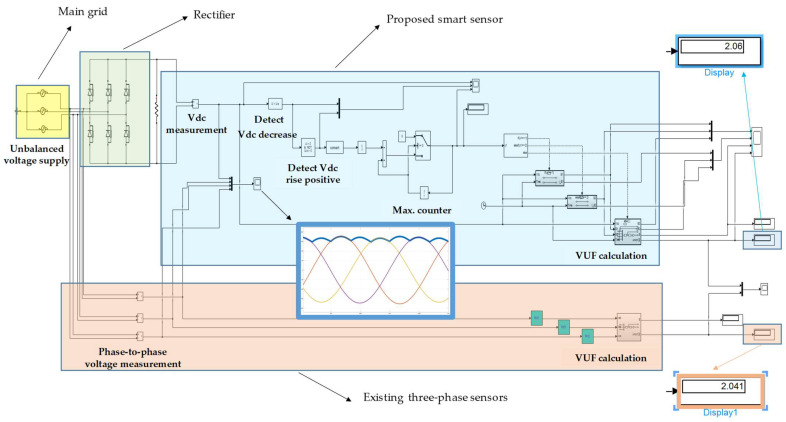
VUF calculation by simulation in Matlab-Simulink^TM^ with the proposed smart sensor and with the existing three-phase sensors.

The proposed methodology in Section 3 has been validated by means of Matlab-Simulink^TM^ software. Figure 5 shows the scheme that has been built in that software, which consists of:

1. Main grid with rated values 100 V and 50 Hz. The following unbalanced scenario in supplied voltages by the grid has been simulated: *V*_a_ = 105 V, *V*_b_ = 100 V, *V*_c_ = 95 V.
2. Three-phase diode bridge rectifier.
3. Proposed smart sensor, where the methodology explained in Section 3 has been implemented in Matlab-Simulink^TM^ blocks. Note that the proposed smart sensor only measures the DC link, unlike existing sensors, which measure three voltages (either phase-to-phase or phase-to-neutral voltages). The VUF has been calculated according to Equations (3) and (4) (see the proposed methodology in Section 3).
4. Existing three-phase sensors, which measure phase-to-phase voltages. The VUF has been calculated according to Equations (3) and (4).

Note from Figure 5 that the proposed smart sensor simulated in Matlab-Simulink^TM^ consists of the following blocks: voltmeter (to measure the DC voltage); detect decrease (to detect the time intervals when the DC voltage decreases); detect rise positive (to detect the positive rise of the DC voltage, which is used to detect the maximum values of the DC voltage); maximum counter (which is used to count the number of local maximums of the DC voltage: note that if 3 maximum values are reached, then the semi-period is obtained, according to Figure 3, and the counter is reset to 0; and “if” blocks (to obtain the *V*_max_ values in groups of 3 in order to calculate the VUF, according to Equations (13) and (14)). So, the input of the smart sensor simulated in Matlab-Simulink^TM^ is the DC voltage and its output is the unbalance results (VUF quantification), which corresponds to the smart sensor concept shown in Figure 1.

Judging by the results given by the simulation in Matlab-Simulink^TM^, it can be concluded that the proposed smart sensor gives a realistic value for the VUF in the simulated unbalanced scenario, i.e., VUF = 2.06%, compared to the value given by existing three-phase sensors, i.e., VUF = 2.041%. Moreover, the authors consider that the VUF given by the proposed smart sensor is even more realistic than the VUF given by existing three-phase sensors, because only one sensor is used, so the introduced measurement error is lower, as will be shown in the experimental results of the next subsection.

### 4.2. Experimental Results

**Figure 6:**
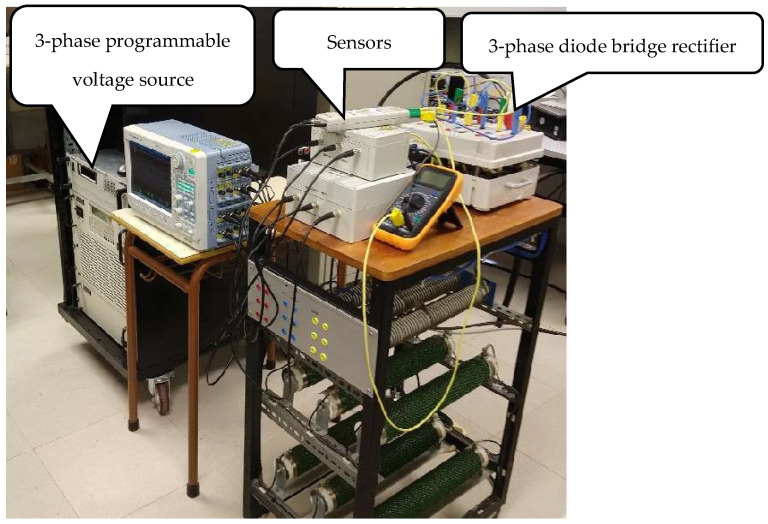
Experimental setup used to validate the proposed methodology.

The experimental validation of the proposed methodology explained in Section 3 has been carried out in the laboratory setup, shown in Figure 6. It consists of a 4.5-kVA three-phase Pacific^TM^ Power Source (model 345AMXT), by means of which the unbalance voltage scenarios have been programmed, and a three-phase diode bridge rectifier (diode modules from Semikron^TM^, model SKKD 46/16) where the voltage measurements have been conducted. The rated voltage (RMS value of the pre-unbalance phase-to-neutral voltage) and the rated frequency have been set to 100 V and 50 Hz, respectively. The following two unbalance voltage scenarios have been considered:

1. Case 1: one-phase voltage drop of 5% (phase a) with respect to its rated value (i.e., *h* = 0.95), which corresponds to the first case shown in Table 1 and Table 2 (see voltage phasors of their first row).
2. Case 2: two-phase voltage drop of 10% (phases b and c) with respect to its rated value (i.e., *h* = 0.9), which corresponds to the second case shown in Table 1 and Table 2 (see voltage phasors of their second row).

**Figure 7:**
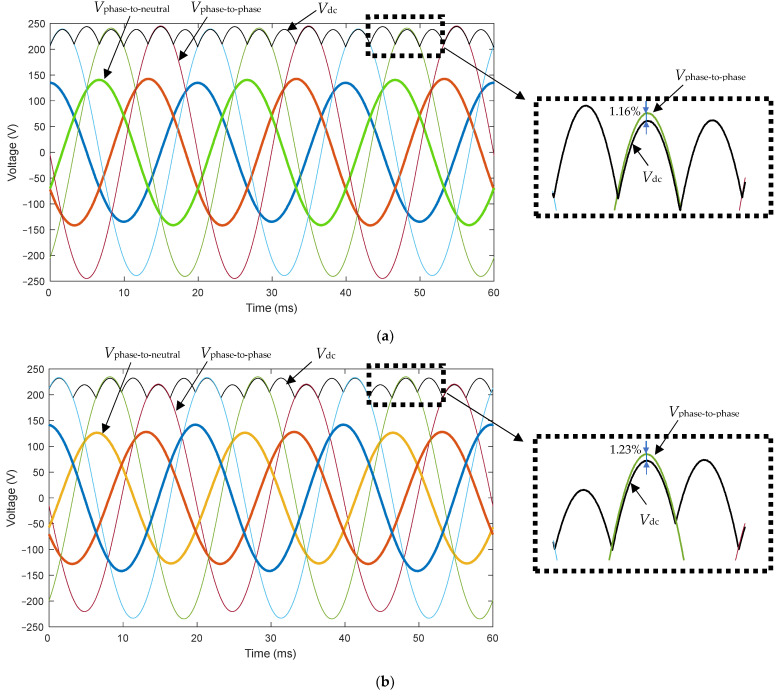
Experimental results. (**a**) Case 1: one-phase voltage drop of 5% (phase a) with respect to its rated value (*h* = 0.95); (**b**) two-phase voltage drop of 10% (phases b and c) with respect to its rated value (*h* = 0.9). Legend: solid thick line (blue, red, orange) = measured phase-to-neutral voltages; solid thin line (blue, red, green) = measured phase-to-phase voltages; solid black line = measured DC voltage.

Figure 7 displays the time evolution of the following measured voltages in the experimental setup: phase-to-neutral voltages, phase-to-phase voltages and DC voltage for the aforementioned Case 1 (Figure 7a) and Case 2 (Figure 7b). It is observed that:

(21) $$V_{\mathrm{dc}}=\max\left\{\left|V_{\mathrm{ab}}\right|,\left|V_{\mathrm{bc}}\right|,\left|V_{\mathrm{ca}}\right|\right\},$$

which is somewhat expected in a three-phase diode bridge rectifier, since its DC output voltage corresponds to the maximum value of the three input AC phase-to-phase voltages at any given time.

The following observation should be pointed out regarding the zoomed region shown in Figure 7: there is a voltage measurement deviation between the phase-to-phase voltages and the measured DC voltage, which can be quantified as:

(22) $$\mathrm{Voltage}\;\mathrm{measurement}\;\mathrm{deviation}\;\left(\%\right)=\frac{\left|V_{\mathrm{phase}-\mathrm{to}-\mathrm{phase}\;\max}-V_{\mathrm{dc}\;\max}\right|}{\left|V_{\mathrm{phase}-\mathrm{to}-\mathrm{phase}\;\max}\right|}\cdot 100,$$

leading to a voltage measurement deviation of 1.16% for Case 1 and 1.23% for Case 2. Consequently, it has been proved that sensors introduce measurement errors; therefore, the more measuring devices we use, the higher the measurement error is. In this regard, it should be noted that the methods in the technical literature propose measuring either phase-to-phase or phase-to-neutral voltages (i.e., using three-phase sensors) to quantify the VUF, while this paper suggests using just one voltage sensor, which measures the DC voltage. Then, the authors consider that the proposed smart sensor to quantify the VUF gives a more accurate value than the existing three-phase sensors, as the measurement error is lower, since only one voltage sensor is needed. Moreover, the international standard IEC 61000-4-30 [6] states that the unbalance factor must be calculated with an uncertainty less than ±0.15% for a class A measuring devices, and less than ±0.3% for class S measuring devices with a measuring range from 1% to 5%; thus, the proposed smart sensor is able to calculate the VUF with less uncertainty than exiting three-phase sensors.

**Figure 8:**
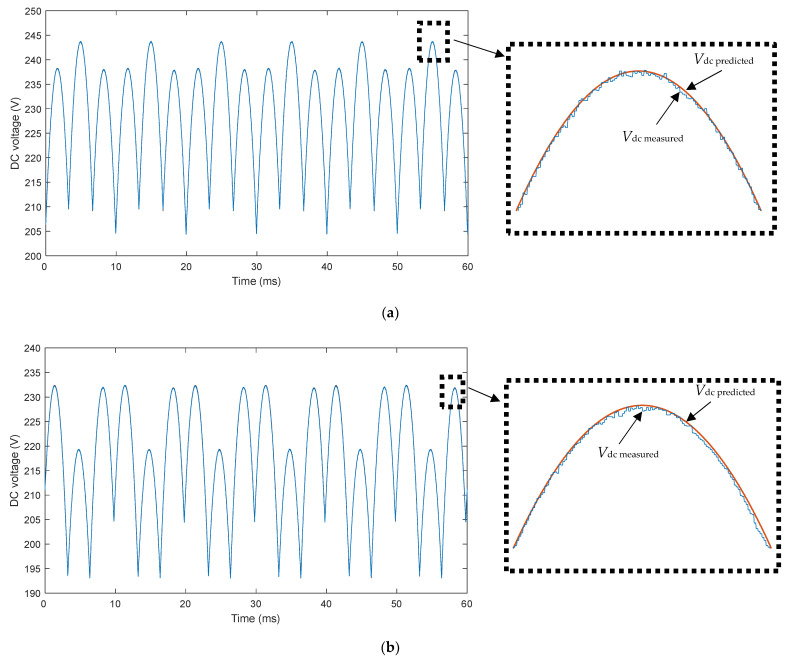
Measured DC voltage and predicted DC voltage by the smart sensor. (**a**) Case 1: one-phase voltage drop of 5% (phase a) with respect to its rated value (*h* = 0.95); (**b**) two-phase voltage drop of 10% (phases b and c) with respect to its rated value (*h* = 0.9). Legend: solid blue line: measured DC voltage; solid red line = predicted DC voltage by the smart sensor.

Figure 8 shows the time evolution of the measured DC voltage and the one predicted by the smart sensor according to the methodology exposed in Section 3 for the aforementioned Case 1 (Figure 8a) and Case 2 (Figure 8b). This figure shows that the *V*_dc_ predicted by the evaluation unit of the proposed smart sensor perfectly follows the waveform drawn by the *V*_dc_ measured by the voltage sensor at the output of the rectifier fed by unbalanced voltages. Consequently, the proposed methodology explained in Section 3 has been successfully validated by experimental tests.

## 5. Proposal of a New Voltage Unbalance Factor

The methodology proposed in Section 3 suggested determining the local minimum values of the DC voltage and their time instants in order to quantify the voltage unbalance factor (VUF). According to this idea, a new voltage unbalance factor is proposed. The authors suggest the following name: VUFT, which means “Voltage Unbalance Factor according to Time”. Indeed, the proposed VUFT only makes use of time instants, unlike the existing methods in the literature, which make use of the measured AC voltages, as discussed in Section 2.

The proposed VUFT consists of obtaining the deviation between the third part of the semi-period and the maximum time lapse between minimum values in DC voltage (see Figure 3 to understand the relation between such time intervals), as follows (in per unit):

(23) $$\mathrm{VUFT}=\frac{\max\left\{t_{\mathrm{dif}\;1},t_{\mathrm{dif}\;2},t_{\mathrm{dif}\;3}\right\}-T_{\mathrm{SP}}/3}{T_{\mathrm{SP}}/3},$$

where *T*_SP_ is the semi-period, which is obtained by means of Equation (11), and *t*_dif 1_, *t*_dif 2_ and *t*_dif 3_ are calculated as:

(24) $$\begin{array}{c} t_{\mathrm{dif}\;1}=t_{\min2}\left(M_{i}\right)-t_{\min1}\left(M_{i}\right)\;\\ t_{\mathrm{dif}\;2}=t_{\min3}\left(M_{i}\right)-t_{\min2}\left(M_{i}\right)\;\\ t_{\mathrm{dif}\;3}=t_{\min4}\left(M_{i}\right)-t_{\min3}\left(M_{i}\right), \end{array}$$

with *M*_i_ being the measurement *M*_1_, *M*_2_, …, *M*_10_ (see Table 3). Note that if the VUFT is given in %, then Equation (23) must be multiplied by 100.

**Figure 9:**
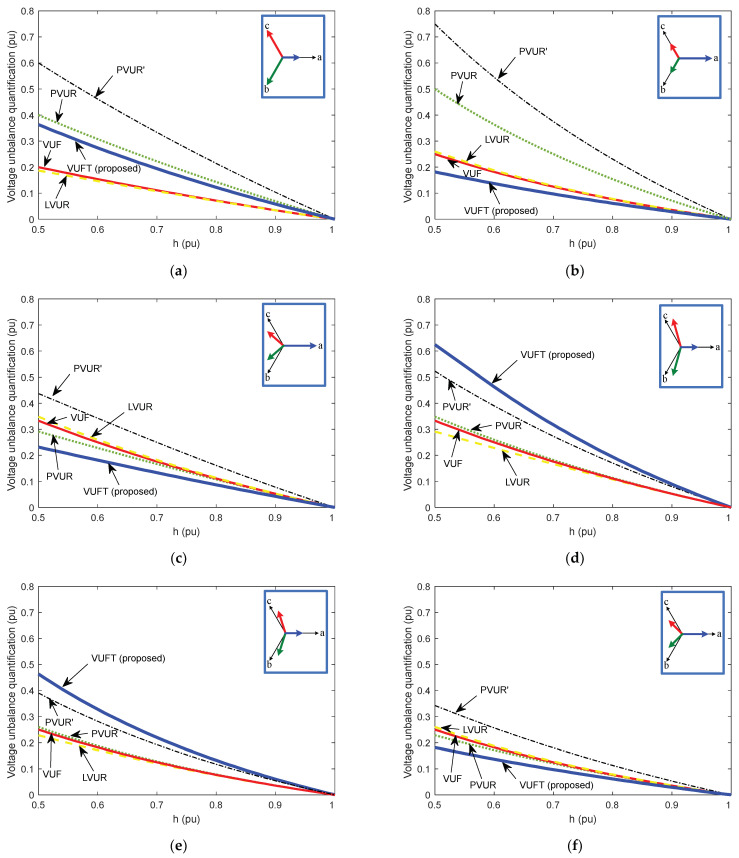
Voltage unbalance quantification (in per unit) by means of the proposed factor (VUFT) and the existing factors in the literature (VUF, LVUR, PVUR and PVUR’). (**a**–**f**) Unbalance voltage scenarios (whose voltage phasors are shown in each subplot). h = remaining voltage with respect to rated voltage (1 = no voltage drop; 0.5 = 50% voltage drop). Legend: solid thick line = VUFT (proposed); solid thin line = VUF; dashed line = LVUR; dotted line = PVUR; dashed-dotted line = PVUR’.

Figure 9 shows the comparison between the proposed VUFT, given by Equation (23), and the existing factors in the literature, which were discussed in Section 2, namely: VUF (Equation (1), according to EN 50160 [1]); LVUR (Equation (5), according to NEMA MG1 [7]); PVUR (Equation (7), according to IEEE Std. 141 [29]); and PVUR’ (Equation (9), according to IEEE Std. 936 [30]). The six unbalance voltage scenarios discussed in Section 2.5 (see Table 1 and Table 2) are considered for the comparison. The quantification of the voltage unbalance is given per unit and the following range is considered for the voltage drop: *h* = 0.5…1 (with 1 being no voltage drop and 0.5 being 50% voltage drop). It is observed that the VUFT values for the different unbalance voltage scenarios are quite close to the values given by the other methods. For example, the unbalanced factors are quantified for a voltage drop of 0.5 pu in the phase voltage corresponding to the a-phase. In this case, the values obtained for the existing factors in the literature are the following: VUF = 0.200, LVUR = 0.187, PVUR = 0.400, PVUR’ = 0.600; while the value of the proposed factor is VUFT = 0.363.

Moreover, it is observed from Figure 9 that voltage unbalance factors based on phase-to-phase voltage may coincide for different scenarios (VUF) or present low variation (LVUR), which is not desirable for calculating the voltage unbalance correctly, as they should have different values for different scenarios. Moreover, the voltage unbalance factors based on the phase-to-neutral voltages (PVUR and PVUR’) have the drawback that these voltages could be easily measured if there is a neutral. However, the proposed VUFT has the following advantages: it is easy to measure (only DC voltage measurement is needed) and it presents a wider range for different voltage unbalance scenarios, making it easier to predict the exact voltage unbalance case. Moreover, it should be noted that this voltage unbalance factor is easy to measure and provides high accuracy, considering that 20 µs sampling periods could be achieved.

## 6. Conclusions

This paper has proposed a new smart sensor to monitor a three-phase installation to detect unbalance voltages. This smart sensor measures the DC voltage to determine the voltage unbalance factor, unlike existing sensors, which measure phase-to-neutral or phase-to-phase voltages to calculate it. A reduction in the number of voltage sensors is achieved, as well as an improvement of accuracy, since only one sensor is used to detect the unbalance between voltages, instead of the usual three sensors. A new methodology, based on the measured DC voltage, with its local maximum and minimum values and their corresponding times, has been proposed to quantify the voltage unbalance. Moreover, a deep analysis of usual unbalances (given by standards) and their comparison has been made. Discrepancies between them have been found in this paper.

Furthermore, a new voltage unbalance factor, named VUFT, has been proposed. This acronym means “Voltage Unbalance Factor according to Time” as it uses time instants (specifically the deviation between the third part of the semi-period and the maximum time lapse between minimum values in DC voltage measurement), unlike the existing methods in the literature, which make use of the measured AC voltages. This voltage unbalance factor is easy to measure and provides high accuracy, considering that 20 µs sampling periods could be achieved. Finally, the proposed methodology has been validated through simulation and experimental results.

## Figures and Tables

**Table 1:** Voltage unbalance quantification by using symmetrical components.

Voltage Unbalance	Phasor Expressions	Positive Seq. ^1^	Negative Seq. ^1^	VUF ^2^
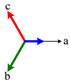	$\begin{array}{l} {\bar{V}}_{a}=h\bar{V}\\ {\bar{V}}_{b}=-\left(1/2\right)\bar{V}-j\left(\sqrt{3}/2\right)\bar{V}\\ {\bar{V}}_{c}=-\left(1/2\right)\bar{V}+j\left(\sqrt{3}/2\right)\bar{V} \end{array}$	${\bar{U}}_{1}=\frac{2+h}{3}\bar{V}$	${\bar{U}}_{2}=-\;\frac{1-h}{3}\bar{V}$	$\mathrm{VUF}=\;\frac{\;1-h}{2+h}$
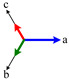	$\begin{array}{l} {\bar{V}}_{a}=\bar{V}\\ {\bar{V}}_{b}=-\left(1/2\right)h\bar{V}-j\left(\sqrt{3}/2\right)h\bar{V}\\ {\bar{V}}_{c}=-\left(1/2\right)h\bar{V}+j\left(\sqrt{3}/2\right)h\bar{V} \end{array}$	${\bar{U}}_{1}=\frac{1+2h}{3}\bar{V}$	${\bar{U}}_{2}=\frac{1-h}{3}\bar{V}$	$\mathrm{VUF}=\;\frac{\;1-h}{1+2h}$
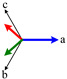	$\begin{array}{l} {\bar{V}}_{a}=\bar{V}\\ {\bar{V}}_{b}=-\left(1/2\right)\bar{V}-j\left(\sqrt{3}/2\right)h\bar{V}\\ {\bar{V}}_{c}=-\left(1/2\right)\bar{V}+j\left(\sqrt{3}/2\right)h\bar{V} \end{array}$	${\bar{U}}_{1}=\frac{1+h}{2}\bar{V}$	${\bar{U}}_{2}=\frac{1-h}{2}\bar{V}$	$\mathrm{VUF}=\;\frac{\;1-h}{1+h}$
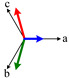	$\begin{array}{l} {\bar{V}}_{a}=h\bar{V}\\ {\bar{V}}_{b}=-\left(1/2\right)h\bar{V}-j\left(\sqrt{3}/2\right)\bar{V}\\ {\bar{V}}_{c}=-\left(1/2\right)h\bar{V}+j\left(\sqrt{3}/2\right)\bar{V} \end{array}$	${\bar{U}}_{1}=\frac{1+h}{2}\bar{V}$	${\bar{U}}_{2}=-\;\frac{1-h}{2}\bar{V}$	$\mathrm{VUF}=\;\frac{\;1-h}{1+h}$
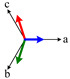	$\begin{array}{l} {\bar{V}}_{a}=h\bar{V}\\ {\bar{V}}_{b}=-\left(1/2\right)h\bar{V}-j\left[\left(2+h\right)/\sqrt{12}\right]\bar{V}\\ {\bar{V}}_{c}=-\left(1/2\right)h\bar{V}+j\left[\left(2+h\right)/\sqrt{12}\right]\bar{V} \end{array}$	${\bar{U}}_{1}=\frac{1+2h}{3}\bar{V}$	${\bar{U}}_{2}=\frac{1-h}{3}\bar{V}$	$\mathrm{VUF}=\;\frac{\;1-h}{1+2h}$
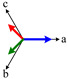	$\begin{array}{l} {\bar{V}}_{a}=[(2+h)/3]\bar{V}\\ {\bar{V}}_{b}=-[\left(2+h\right)/6]\bar{V}-j\left(\sqrt{3}/2\right)h\bar{V}\\ {\bar{V}}_{c}=-[\left(2+h\right)/6\bar{V}+j\left(\sqrt{3}/2\right)h\bar{V} \end{array}$	${\bar{U}}_{1}=\frac{1+2h}{3}\bar{V}$	${\bar{U}}_{2}=\frac{1-h}{3}\bar{V}$	$\mathrm{VUF}=\;\frac{\;1-h}{1+2h}$

^1^ Obtained by applying the *Fortescue* transformation [28], i.e., Equation (2), to the phasor expressions. ^2^ According to EN 50160 [1], i.e., by means of Equation (1) in per unit. *h* = remaining voltage with respect to rated voltage (1 = no voltage drop; 0 = 100% voltage drop).

**Table 2:** Voltage unbalance quantification by using phase-to-phase (line) voltages or phase-to-neutral voltages.

Voltage Unbalance	LVUR ^1^	PVUR ^2^	PVUR’ ^3^
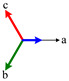	$\frac{3\left(2\sqrt{3\left(h^{2}+h+1\right)}-3\right)}{{\left(2h+1\right)}^{2}}-1$	$\frac{6\left(h-2\right)}{h^{2}-4}-2$	$\frac{9\left(h-2\right)}{h^{2}-4}-3$
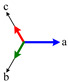	$\frac{3\left(3h^{2}-2h\sqrt{3\left(h^{2}+h+1\right)}\right)}{{\left(h+2\right)}^{2}}+1$	$\frac{3}{2h+1}-1$	$\frac{18h-9}{8h^{2}-2}-\frac{3}{2}$
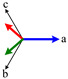	$h\left(h-\sqrt{\left(h^{2}+3\right)}\right)+1$	$\frac{\sqrt{3h^{2}+1}-1}{h^{2}}-1$	$\frac{3\left(\sqrt{3h^{2}+1}-1\right)}{2h^{2}}-\frac{3}{2}$
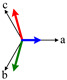	$\frac{\sqrt{\left(3h^{2}+1\right)}-1}{h^{2}}-1$	$h\left(h-\sqrt{h^{2}+3}\right)+1$	$\frac{3h\left(h-\sqrt{h^{2}+3}\right)}{2}+\frac{3}{2}$
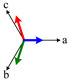	$\frac{2\left(-2h+\left(h+2\right)\sqrt{\left(7h^{2}+h+1\right)}-2\right)}{9h^{2}}-\frac{10}{9}$	$\frac{3\left(3h^{2}-2h\sqrt{3\left(h^{2}+h+1\right)}\right)}{{\left(h+2\right)}^{2}}+1$	$\frac{9h\left(3h-2\sqrt{3\left(h^{2}+h+1\right)}\right)}{2{\left(h+2\right)}^{2}}+\frac{3}{2}$
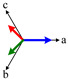	$\frac{3\left(3h^{2}-2h\sqrt{3\left(h^{2}+h+1\right)}\right)}{{\left(h+2\right)}^{2}}+1$	$\frac{\left(h+2\right)\left(2\sqrt{7h^{2}+h+1}-\left(h+2\right)\right)}{9h^{2}}-1$	$\frac{\left(h+2\right)\left(2\sqrt{7h^{2}+h+1}-\left(h+2\right)\right)}{6h^{2}}-\frac{3}{2}$

^1^ According to NEMA MG1 [7], i.e., by means of Equation (5) in per unit. ^2^ According to IEEE Std. 141 [29], i.e., by means of Equation (7) in per unit. ^3^ According to IEEE Std. 936 [30], i.e., by means of Equation (9) in per unit. *h* = remaining voltage with respect to rated voltage (1 = no voltage drop; 0 = 100% voltage drop).

**Table 3:** Ten measurements of 3 consecutive maximum values of the DC voltage and 4 consecutive time values in which the DC voltage has its minimum values. The values correspond to the DC voltage profile shown in Figure 3.

Meas. ^1^	*t*_min 1_ (ms)	*V*_max 1_ (V)	*t*_min 2_ (ms)	*V*_max 2_ (V)	*t*_min 3_ (ms)	*V*_max 3_ (V)	*t*_min 4_ (ms)
*M*_1_	3.36	243.69	6.65	237.88	9.94	238.27	13.36
*M*_2_	13.36	243.73	16.65	238.04	19.94	238.27	23.36
*M*_3_	23.36	243.73	26.65	237.92	29.94	238.25	33.36
*M*_4_	33.36	243.75	36.65	238.04	39.94	238.27	43.36
*M*_5_	43.36	243.75	46.65	237.9	49.94	238.27	53.36
*M*_6_	53.36	243.71	56.65	237.9	59.94	238.31	63.36
*M*_7_	63.36	243.69	66.65	237.94	69.94	238.38	73.36
*M*_8_	73.36	243.67	76.65	238.00	79.94	238.27	83.36
*M*_9_	83.36	243.71	86.65	237.92	89.94	238.29	93.36
*M*_10_	93.36	243.75	96.65	238.04	99.94	238.29	103.36

^1^ This table is fully updated with 10 new measurements every 5 cycles.

## List of Abbreviations

AC	Alternating current.
DC	Direct current.
LVUR	Line voltage unbalance ratio (according to NEMA MG1).
PCC	Point of common coupling.
PVUR	Phase voltage unbalance ratio (according to IEEE Std. 141).
PVUR’	Phase voltage unbalance ratio (according to IEEE Std. 936).
RMS	Root mean square.
VUF	Voltage unbalance factor (according to EN 50160).
VUF’	Voltage unbalance factor (according to CIGRE and IEEE Std. 1159).
VUFT	Voltage unbalance factor according to time (proposed factor).
